# ADHD and reification: Four ways a psychiatric construct is portrayed as a disease

**DOI:** 10.3389/fpsyt.2022.1055328

**Published:** 2022-12-14

**Authors:** Sanne te Meerman, Justin E. Freedman, Laura Batstra

**Affiliations:** ^1^Department of Child and Family Welfare, University of Groningen, Groningen, Netherlands; ^2^Department of Interdisciplinary and Inclusive Education, Rowan University, Glassboro, NJ, United States

**Keywords:** ADHD, reification, critical discourse analysis, epistemic violence, genetic reductionism, ecological fallacy

## Abstract

**Introduction:**

The descriptive classification Attention-Deficit/Hyperactivity Disorder (ADHD) is often mistaken for a disease entity that explains the causes of inattentive and hyperactive behaviors, rather than merely describing the existence of such behaviors. The present study examines discourse on ADHD to analyze how authors passively and actively contribute to reification—a fallacy in which a concept is represented as a thing existing on its own.

**Methods:**

Critical Discourse Analysis and Qualitative Content Analysis of academic textbooks, scientific articles, websites and videos were used to analyze how ADHD is reified.

**Results:**

The analyses reveal four ways in which inattentive and restless behaviors are presented as an entity by means of the ADHD classification: language choice, logical fallacies, genetic reductionism, and textual silence. First, language choice, such as medical jargon and metaphors aid in representing ADHD as a disease entity. Second, several logical fallacies do the same, including the relatively unknown “ecological fallacy” that refers to the erroneous belief that average group findings, such as average brain size of groups of those with an ADHD classification, can be applied on an individual level. Third, genetic reductionism is often achieved by overstating the results of twin studies and being silent about the disappointing molecular genetic research. Such textual silence is the last identified mechanism of reification and includes instances in which societal factors that affect the ADHD construct are often omitted from texts, thereby obscuring the extent to which ADHD is a limited heuristic.

**Discussion:**

It is essential that discourse communities do not repeat these four ways of reifying behavior and social relations into an alleged entity with the acronym ADHD. The errors and habits of writing may be epistemologically violent by influencing how laypeople and professionals see children and ultimately how children may come to see themselves in a negative way. Beyond that, if the institutional world shaped to help children is based on misguided assumptions, it may cause them harm and help perpetuate the misguided narrative. To counter the dominant, reifying and medicalizing view, guidelines such as the recently published “Dutch ADHD Psychoeducation Guidelines” might be helpful.

## Introduction

In a study of children’s views on Attention-Deficit/Hyperactivity Disorder (ADHD), Singh (1) reports that 11-year-old Sylvia believes that “ADHD is like a cancer disease but you’re not going to die from it” (p. 20). In another study, a young person (age unspecified) stated “it’s like a disease eating on you” [(2), p. 207].

These are troubling, but not uncommon, examples of the “reification” of ADHD. Reification literally means “to make” (from the Latin *facere*) a “thing,” (from the Latin word “*Res*”). The children quoted above seem to understand ADHD as a thing, a concrete disease entity inside their bodies. Reification, also known as a “fallacy of misplaced concreteness” (3), is a longstanding and problematic phenomenon. For example, in their sociological classic *The social construction of reality* Berger and Luckmann (4) forewarn that abstract concepts and social phenomena are in danger of being reified “even if it begins by modestly assigning to its constructs merely heuristic status” [(4), p. 208]. In this paper, we are concerned precisely with how the heuristic construct of ADHD is “reified” through certain linguistic patterns and logical fallacies in discourse and increasingly represented as an entity existing in nature.

### Attention-Deficit/Hyperactivity Disorder: A heuristic construct

To explain ADHD is not at all comparable to a concrete disease like cancer, but is in fact a heuristic construct, the best point of departure is likely the Diagnostic and Statistical Manual of Mental Disorder (DSM) itself; the psychiatric handbook that defines all psychiatric disorders. The fourth edition in particular is conscious of its limitations. The guidebook states that most of the classifications in the DSM are exactly that: “valuable heuristic constructs” instead of “well-defined entities that describe nature exactly as it is” [(5), p. 12]. To be eligible for an ADHD classification a child must meet 6 out of 9 behavioral criteria for inattention, such as “often fails to give close attention to details or makes careless mistakes” and “often avoids, dislikes or is reluctant to engage in tasks that require sustained mental effort (…)” and/or 6 out of 9 criteria for hyperactivity such as: “often unable to play or engage in leisure activities quietly.” Besides the risk of pathologizing personal preferences—not every child likes concentrating on scholastic tasks and some children enjoy being vocal more than others during play—the criteria are also highly sensitive to subjectivity, with each containing the adverb “often.” A diagnostic classification requires meeting several other criteria, such as the “age of onset” criterion. It is noteworthy that the age of onset is raised from 7 in the DSM-IV American Psychiatric Association (APA) (6) to 12 in the DSM 5 APA (7). Children of prepubertal age are therefore also eligible to be classified now (8). Additionally, there is an ‘impairment criterion’ in the DSM 5, which stipulates “symptoms interfere with, or reduce the quality of, social, academic, or occupational functioning” [(7), p. 60]. The standard is lowered compared to the DSM-IV that urged for “Clear evidence of clinically significant impairment in social, academic, or occupational functioning” [(6), p. 93]. Finally, children must fit these criteria in at least two different settings.

### Reification in mental healthcare

Fortunately, the problem of the reification of the DSM’s heuristic constructs is occasionally addressed. For instance, Hyman (9), the former director of the National Institute of Mental Health (NIMH), quotes 19th century Philosopher John Stuart Mill to explain the problem of reification in mental healthcare: “The tendency has always been strong to believe that whatever received a name must be an entity or thing, having an independent existence of its own” (p. 46).

Hyman asserts that when sets of behavioral and additional criteria receive a name, such as ADHD, they are commonly misinterpreted as discrete entities. Such confusion is comparable to what logicians describe as the “nominal fallacy.” By naming a certain phenomenon, such as a set of behaviors, we falsely believe that we have thereby explained it. Hyman states that reification is a problem (partly) related to the use of language in mental healthcare. Language that: “if unchanged (…) will further calcify what I argue is a highly problematic *status quo*” [(9), p. 159]. This *status quo* includes, amongst others, the omnipresent, yet stigmatizing and erroneous suggestion that we know about an *individual’s* brain *abnormalities* based on small *group differences*.

However, Hyman does not explicate beyond the “nominal fallacy” to describe exactly how language affects reification. The present research aims to address the issue of reification in mental healthcare by examining the construct of ADHD. This study begins with the research question: what discursive elements reify the behaviors that fall under the ADHD classification?

## Materials and methods

Critical Discourse Analysis (CDA) offers a suitable theoretical framework for answering the above research question as it is concerned with both discourse and reification. CDA mostly relates reification to nominalization [see e.g., (9, 10)] but other discursive elements studied within CDA, such as generalizations, are related to reification as well (11). To study how these linguistic elements reify, we use Qualitative Content Analysis (QCA). QCA is useful here as it allows for theoretically driven, deductive coding. At the same time, as theory on how exactly discourse reifies is not abundant, QCA has “emergent flexibility” to allow data-driven codes to emerge, changing the coding frame, additional data to be gathered which facilitates a cyclic process of going back and forth between data and theory. This method also allows concepts from other qualitative methods to be integrated in its (deductive) coding and QCA explicitly mentions concepts within CDA for this purpose (12).

As qualitative content analysis does not take a clear epistemological stance, it seems important to clarify ours. Our research is rooted in “weak constructionism,” which assumes that a certain phenomenon, such as behaviors that are interpreted within a given cultural context, can have a material basis (e.g., brain processes). Yet, the manner in which people discuss such a phenomenon is socially constructed (13). In the present study, we are not only interested in the way people construct discourse surrounding the alleged material basis, but also with the ontological realness of the “thing”—ADHD—and how in several instances its ontology is predicated on scientific and logical errors in interpreting the available empirical data. We analyze literature that exemplifies flaws in validity, reliability and logic in the way empirical research is represented.

### Data selection

This is a critical review, which differs from e.g., a systematic review by primarily intending to spark a conceptual discussion more than pretending to have a systematic and quantifiable empirical base [see e.g., (14)], on which for instance claims about the prevalence of certain discursive elements can be made. In other words, we primarily set out to create an overview of different linguistic devices that reify ADHD. Only occasionally, when data is available, do we make statements about an estimated incidence of such reifying mechanisms. For our data collection, we have used “purposeful sampling” (15): we included information rich sources on reifying mechanisms in discourse on ADHD. Our criterion to complete the gathering of data was based on “saturation” [(16), p. 38] which means new data was added to the sample until no new additional mechanisms of reification were found. For this we initially used the data of earlier studies into academic textbooks and scientific statements about ADHD [e.g., (17, 18)] which we have expanded with examples found on websites, in video’s on ADHD and in ADHD related research articles.

## Analysis

Our analysis revealed roughly four ways in which the abstract DSM-definition of ADHD is reified into a concrete disease: language choice, logical fallacies, genetic reductionism, and textual silence. Table 1 summarizes these four main themes using examples from the data. Next to naming the mechanisms of reification, we describe our preferred ways of communicating about ADHD.

**TABLE 1:** Examples of four categories of reification.

Reifying words/phrases	Preferred way	Explanation
***1. Language choice***		
Symptom/diagnosis	Criteria	Symptom refers to ‘’evidence of disease,” while criterion, “a standard on which a judgment is based” exposes normativity and subjectivity.
Diagnosis	(Behavioral) Classification	Diagnosis refers to identification of disease, while classification refers to grouping based on (behavioral) similarities, which is more factual.
Children with ADHD	Children who behave hyperactive or inattentive or meet criteria according to a professional	Nominalization and passive phrasing removes agency from children and can often be avoided.
***2. Logical fallacies***		
ADHD affects educational and occupational performance	Problems with educational/occupational performance are part and parcel of the ADHD construct	Suggesting that ADHD affects educational and occupational performance is circular as compromised performance in these areas are part of the criteria for a diagnosis.
Families with adolescents who have both ADHD and oppositional disorder appear to have more than the usual number of arguments, negative communications, and hostility.	When adolescents have more than the usual number of arguments, negative communications, and hostility, they could be classified with both ADHD and oppositional disorder.	The circularity can be avoided by showing the logical order of things: people are classified because of their behaviors. In writing, the behaviors would preferably precede the classification as well.
***3. Genetic determinism***		
Research shows that among children at high risk for ADHD, positive parenting can provide a buffer.	Research shows that outgoing children are at risk for developing problematic behavior in adverse circumstances while positive parenting can reduce the risk.	We should avoid suggesting an innate genetic disorder and framing these behaviors as something that needs a *buffer* against when these behaviors have not yet developed into problematic behaviors and both positive parenting and adverse circumstances can influence the development of children and their behavior for better or worse.
Several genes are involved with ADHD.	Several genes are associated with ADHD, although they explain little of the behaviors. Many people who behave unruly/inattentive do not have those genes while many people without attentional difficulties can have these genes as well.	Mere involvement of genes is too vague, as they explain little of a child’s behavior according to empirical findings. Being more explicit about effect size –in understandable language- helps to avoid overstating the impact of genes.
***4. Textual silence***		
ADHD places an economic burden on families and society	ADHD has many different causes -including societal demands, poverty, overcrowded classrooms, etc. which can place a financial burden on the medical system if not tackled from a broader perspective like poverty policy and investing in education.	Many crucial topics are often left out, with the ‘cost of ADHD’ narrative as an example. For instance, birth-month studies show the youngest in class are often given a ‘diagnosis’ of ADHD and receive stimulants for their age-appropriate behavior. The ‘costs’ of ADHD are also comprised by such misapplication of our medical approach but these and other societal influences are obscured by the economic burden of the ADHD narrative.

## Language choice

The assertion that language choice is important in mental health-care is not new. (Szasz (19), cover text) argued that “psychiatrists have misapplied the vocabulary of disease.” More recently, in their paper “Drop the language of disorder,” Kinderman et al. (20) plead for careful consideration of our vocabulary when describing psychological distress to avoid framing normal reactions to circumstances as indications of pathology. In what follows, we discuss how some scholars who write about ADHD use nouns, metaphors and acronyms in ways that reify ADHD as a brain disorder.

### Nouns

Reification is facilitated by transforming complex human (inter)actions into discrete or countable entities in the form of nouns (21, 22). In the case of ADHD, the use of nouns such as “diagnosis” and “symptoms” contribute to reification. For example, the behavioral criteria the DSM lists for ADHD are called: “symptoms.” Clinicians are directed to count these symptoms and when children exhibit 6 out of 9 of them, children become eligible for an ADHD “diagnosis.” For instance, a symptom of hyperactivity and impulsivity is “often runs about or climbs in situations where it is inappropriate” [(7), p. 60]. Like many criteria for an ADHD “diagnosis” in the DSM, this description frames situational actions as medical “symptoms,” thereby obscuring the agency of the individual actors and implying that the (mis)behavior was caused by an underlying brain disorder (23).

While describing behaviors and social relations as neatly countable “symptoms” is reifying in itself, the very meaning of words like “patient,” “diagnosis,” and “symptoms” also contribute to reification. The Merriam Webster dictionary (24) defines a “symptom” as “subjective evidence of disease or physical disturbance.” This suggests that an innate entity causes the behaviors. However, there is no proof for physical ailment with ADHD. As children may have *motives* for standing up in a classroom instead of (physical) causes, medical laden nouns often fall short of representing human behavior, while contributing to reifying these behaviors into medical disease entities. For this reason, Dehue (25) argues that the word “criterion,” also used occasionally in different versions of the DSM, is more appropriate. It denotes “a standard on which a judgment or decision may be based” (24) which makes the agency and subjectivity in the decision process more visible and does not relate it to “evidence” but to a “standard.”

### Metaphors

Metaphors are linguistic devices that can have a powerful reifying effect, particularly when medical scientists use metaphors to construct illness. Metaphors have been frequently used to illustrate ADHD as a harmful brain disorder (26). Barkley (27), an influential proponent of the validity of ADHD as a brain disorder, provides a vivid example of the use of a metaphor to describe ADHD in an address to parents of children identified as having ADHD:

“Now I want you to understand something. Your brain can be split into two pieces. The back part is where you acquire knowledge. The front part is where you use it (…). ADHD, like a meat-cleaver, just split your brain in half.” [(27), 1:17:00]

Barkley uses the “meat-cleaver” metaphor to transform ADHD, a subjective classification of children’s behavior, into a concrete object. The metaphor of a “meat-cleaver” reifies by portraying ADHD as an agent that can split brains. Further, a meat-cleaver arguably has a strong “fear appeal” [(28), p. 4], which can portray ADHD as dangerous, and urge listeners to seek help from medical professionals, as Barkley later urges when discussing treatment for ADHD [(27), 1:18:20]. Danforth and Kim (26) have critiqued Barkley’s persistent use of metaphors for removing the agency of individuals, such as when Barkley describes ADHD as a form of imprisonment of the mind. The meat-cleaver metaphor in particular is a “deceptive metaphor” [(29), p. 151] in that empirical evidence does not support this comparison with ADHD; empirical findings indicate versatile, interacting causes and motives for such behaviors while the molecular-genetic and neuro-anatomical correlations are weak and causality is far from clear (18).

### Acronyms

Attention-Deficit/Hyperactivity Disorder is commonly abbreviated with the acronym ADHD. The use of acronyms is a written convention that demonstrates a preference for brevity, and one that we have adhered to in this paper in our use of “ADHD.” Yet, there are consequences of using such acronyms, as they can obscure processes and choices in the scientific production of knowledge, a process that Latour calls “*blackboxing*” (30). The use of the term ADHD is reifying in that it suggests an entity, as opposed to describing active behaviors. For example, using active phrasing, such as children who *behave hyperactive* or *inattentive* helps to avoid reifying human behavior. The use of active phrasing helps to display agency (21), which reminds us there are children who (mis)behave in a certain manner, according to the perceptions of other individuals. In contrast, an acronym such as “ADHD” nominalizes the actions [(31), p. 148] and reifies by shifting the focus and agency to the named entity, away from the actors and their actions.

Furthermore, beyond individual actions, reification also detracts from attention to social relations, which has been acknowledged from the very inception of the concept (32). In relation to ADHD, it is noteworthy that several of the criteria for an ADHD classification imply a social relation in a particular (school)context. Examples are criterion 1.b/c for hyperactivity: “Often leaves seat in situations when remaining seated is expected” and “often runs about in situations where it is inappropriate” [DSM 5, APA (7)]. Contemporary schools and initiatives experiment with ‘flipping the classroom’ such as improving the possibilities for physical activity in and outside of school (33), and the use of standing desks (34). This implies that being active and not being seated in the classroom are normalized in contexts where it was previously deemed inappropriate and reason for disciplinary measures or considered as a criterion for a psychiatric classification. Hence, such educational innovation exposes the social nature of behaviors that are ‘blackboxed’, reified and pathologized with the ADHD acronym.

## Logical fallacies

### Generalizations

Within scientific literature, findings of group studies about ADHD, such as average differences between the brains of those with and without a classification, are often generalized as if they apply at the individual level [see e.g., (35, 11)]. This generalization has a reifying effect by representing ADHD as an identifiable disease entity existing inside the brains of those with ADHD. For example, in the largest study to date on the neuroanatomy of those classified with ADHD (36) the authors concluded that “the data from our highly powered analysis confirm that patients with ADHD do have altered brains and therefore that ADHD is a disorder of the brain” (p. 316). However, in logical terms, no brain-anatomical feature is *necessary nor sufficient* (37) for an ADHD classification. The small effect sizes of the case-control study in fact reveal that many classified with ADHD *do not* have smaller brains or brain parts than average, so it is not a *necessary* condition. At the same time, many who do not display behaviors that comprise the ADHD criteria do have smaller brains than average, so it is not a *sufficient* condition either. The authors do not address this issue and instead conclude that “This message is clear for clinicians to convey to parents and patients.” The individualized message for “patients”— about their ‘disorder of the brain’ reifies ADHD by making it appear as a discrete attribute—that every individual classified with ADHD has a detectable, physical anatomical feature. This methodological error is known as the ecological fallacy. This fallacy refers to how studies that use aggregate measures—often mean values—of attributes like brain size, are very limited in what they can infer about individuals in the population being studied (38).

In response to this study, critics brought additional methodological concerns to the public’s attention. For instance, the reported effects of the study by Hoogman et al. (36) disappear when controlled for IQ as the case group had lower average IQ than the control group (39). This may be due to biased sampling, common in many case-control MRI studies according to Horga et al. (40). Often *refined phenotypes*, thoroughly screened individuals, are compared to *supernormal* controls (41, 42). These controls are healthier than average and do not accurately represent the “normal” population, which makes the generalization further overstated. Inconsistency through time is also a problem. Hoogman et al. (36) themselves report that these brain differences are not a permanent group feature. On average, brain growth in those classified with ADHD catches up with that of controls, an issue that further draws into question the authors’ claim of ADHD being a neurobiological disorder.

### Brain scans and the risk of the ecological fallacy

**FIGURE 1:**
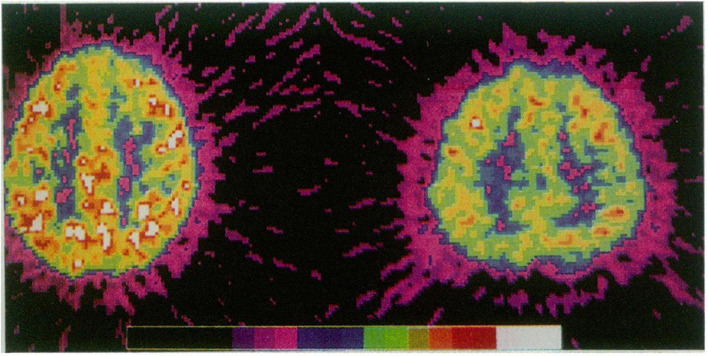
Adapted from ref. (44) Copyright ©1990 of this illustration is retained by the Massachusetts Medical Society.

Studies attempting to draw on neurophysiology (brain activity) and neurochemistry (amount of e.g., neurotransmitters like dopamine) as the basis of the ADHD construct are hampered by all the aforementioned problems: biased sampling, only small group differences, no particular pattern of brain activity being necessary of sufficient for an ADHD classification and inconsistency through time. However, these studies are burdened further by a lack of consistency and reliability than neuro-anatomical studies. Brain activity is highly variable and it is “unknown whether the same findings (…) would be found one hour later, one day later, or one year later following the original scan” [(43), p. 219]. Often, brain activity is presented as a fixed attribute. For instance, Figure 1—dispersed widely on the internet as proof of ADHD, compares two PET scans that suggest that all those with ADHD have a specific pattern of brain activation (44).

What we see on the image are two individuals, one from the case and one from the control group whose brain activity has been measured during certain tasks. The measured numerical values—transferred to colors based on thresholds set by the researchers—display that during the test the two individuals’ brain activities were different (45). However, the foregoing considerations mean that no two measurements, even of the same person, are necessarily alike.

Additionally, for the construction of the characteristic—and widely dispersed—photo-like images of the alleged ADHD brain type it is a standard practice to select extreme examples from the already extreme samples that comprise the case and control groups [(45), p. 200]. These illustrations are reifying by suggesting that a certain activity of the brain is a fixed and unique trait for those with an ADHD classification, despite the fact that the empirical data shows the exact opposite: this brain activity is not fixed and it is not unique to those with an ADHD classification.

### Circular arguments

Another common logical fallacy that can reify ADHD is circular argumentation. For example, Biederman and Faraone (46) state “ADHD affects 8–12% of children worldwide, and results in inattention, impulsivity, and hyperactivity” (p. 237). In the order that the authors describe, it seems as if a discrete medical entity—ADHD—causes (i.e., “results in”) symptoms. In fact, children who display these behaviors can be classified with ADHD because these are the very behaviors that are used to define the disorder. It is circular to suggest that the name for these behaviors is the cause of these behaviors. Such circularity reifies ADHD by suggesting that it is a discrete brain disorder that causes certain unwanted behaviors, rather than a construct that is used to name the behaviors.

Another example of circular arguments occurs when behaviors associated with ADHD are linked to other categories of mental disorders. When two or more categories are used to describe children’s behaviors, through multiple diagnoses, this is commonly referred to as “comorbidity,” a medical term that denotes “Existing simultaneously with and usually independently of another medical condition” (24). ADHD is commonly described as being present along with other mental disorders, such as Oppositional Defiant Disorder (ODD). In research literature, authors sometimes portray the existence of both disorders as being *correlated* to the very behaviors that *define* these disorders. For example, the following example comes from an article in the Journal of Abnormal Child Psychology:

“Parents and teens in the ADHD/ODD group rated themselves as having significantly more issues involving parent–teen conflict, more anger during these conflict discussions, and more negative communication generally, and used more aggressive conflict tactics with each other than did parents and teens in the CC [control] group.” [(47), p. 557]

The researchers’ findings that ADHD and ODD are associated with the behaviors they describe illustrate circular logic. Nieweg [(48), p. 693] refers to such circularity as magically pulling a rabbit from a hat that was already put there. Anger, negative communication and parent-teen conflict can all be expected in those identified with Oppositional and Defiant Disorder as these behaviors strongly resemble the criteria for this classification, such as “is often angry and resentful” (A3), “often argues with authority figures” (criteria A4) [(6), p. 462]. The authors reverse the order of things and suggest that a disorder has caused the behaviors. It would be more logical to suggest that active/inattentive children that are involved in more parent-teen conflict, have more negative interactions and display hostility are at risk for getting classified with both ADHD and ODD in a contemporary psychiatric setting. Framing the relationship in a circular manner instead has the reifying effect of making the behaviors appear to be the result of discrete disorders.

### Correlation and cause

Another common way in which ADHD is reified in literature is by confusing correlation with cause. For example, several studies show that prison inmates are often restless and have attentional problems (49–51). When writing about this phenomenon in scientific journal articles, causality is often implied by suggesting that ADHD poses a risk for later delinquency, such as in the following excerpt from Ginsberg et al. (52):

“Adults with ADHD are at increased risk for unemployment, sick leave, coexisting disorders, abuse, and antisocial behavior leading to conviction (p. 1).”

The suggestion here is: “*Post hoc*, ergo Propter hoc”: after this, therefore because of this (35). This is a fallacy as it jumps to conclusions; there are many confounding variables in the history of delinquents, such as family background (53) and child maltreatment (54), that might explain their problematic behaviors leading to confrontations with legal authorities as well as to the delinquents’ attentional problems and unruly behavior. The restlessness of those committing felonies might be a function of their complicated personal histories while, in addition, their current predicament is far from unproblematic as well.

Besides confusing correlation and cause, relating ADHD to more severe problems like delinquency seems to be a reifying mechanism in itself. For instance, in an academic textbook (55), the section on ADHD highlights the case study of a child as follows:

“Sean [He] would never think before he did stuff. And actually, the thing that really made me go, ‘Something is desperately wrong here’—we had a little puppy. Real tiny little dog. And Sean was upstairs playing with it. And my daughter had gone upstairs, and went, ‘Mom, something’s wrong with the dog’s paw.’ And I looked and this poor little dog had a broken paw. Sean had dropped her. But—didn’t say anything to anyone. Just left the poor little dog sitting there. And I thought, ‘Wow. This is just not normal’.” (p. 517)

In this example, readers have no way of knowing why Sean has not reported about the broken paw. He might not have realized it, he might have felt ashamed that he dropped it by accident, or he might not have cared too much. In any case, it is unclear how this example should clarify anything in relation to ADHD because the behaviors do not represent any of the criteria for ADHD. The passage apparently should attest to the seriousness of ADHD and can reify by emphasizing the abnormality and problematic nature of Sean’s behavior.

When examining how textbooks used in university teacher educator courses depict ADHD, Freedman (17) found similar descriptions, including stories about individuals diagnosed with ADHD who burned down their family’s home, attempted to flush their cat down the toilet, or who had an imaginary friend that was too busy to speak to them. None of those behaviors resemble any of the ADHD criteria, yet expanding descriptions of children to include these stories invokes a sense of seriousness and severity about ADHD, representing it as an urgent problem to be solved.

### Genetic reductionism

The example mentioned earlier by Ginsberg et al. (52) which correlates ADHD and delinquency also uses another form of reification. They begin their statement in which they suggest ADHD can cause delinquency by the following passage “ADHD is a common, inherited and disabling developmental disorder with early onset” (p. 1). This reifying mechanism involves blurring the difference between heritability and inheritance which are in fact two very different concepts. *Heritability—*mostly estimated on the basis of behavioral similarities and differences between identical and fraternal twins, is often claimed to be high. In this case no DNA material whatsoever has been studied. Heritability is not to be mistaken with *inheritance*, based on molecular genetic studies. In such studies, DNA material is analyzed. Inheritance refers to the transfer of genetic information from parents to children. However, as there are no clear genetic markers that can predict ADHD behaviors to any substantial degree, the established inheritance of ADHD is in fact very low. Initially, candidate-gene studies claimed small correlations between some genetic variants and ADHD behaviors. However, the finding of a mere 3.3% variance that seven of the best-established genetic variants could allegedly explain (56) has later been rebuked by Genome Wide Associations Scans (57). These more powerful studies have not been able to establish strong correlations between DNA and ADHD and the role of genetics in ADHD is still elusive.

Unfortunately, the difference between heritability and inheritance is muddled regularly in discourse. An illustrative example comes from a study of academic textbooks used in universities in the Netherlands (18). In roughly half of the textbooks that discuss ADHD, only the relatively high estimates of heritability that stem from twin and family studies—allegedly 70–80% in ADHD—are mentioned. Few authors of academic textbooks admit that when studying inheritance “the search to identify specific genes has been disappointing” [(58), p. 246]. More often, authors are not as forthcoming about the limitations of current research that attempts to link ADHD to certain genes. The fact that high estimates from twin studies are by no means reproduced when studying actual DNA differences between those with and without an ADHD classification, is mentioned only in 25% of the sample of textbooks (18). This issue is known as the “missing heritability problem” (59).

Furthermore, there are in fact many environmental correlates that are much stronger than the weak associations of ADHD and genetic variation. For e.g., Sagvolden et al. (60) report that, “parents of children with ADHD often show conduct problems and antisocial behavior (∼25%), alcoholism (14–25%), histrionic or affective disorder (10–27%), or learning disabilities” [(60), p. 417]. Genetic determinism seems also aided by the suggestion that despite these correlates, the environment is “only contributory” [(61), p. 584] and a positive environment is considered “protective” (against what?) as “research shows that among children at high risk for ADHD, positive parenting can provide a buffer” [(62), p.224].

This is a flawed interpretation of heritability because “both inherited and non-inherited factors contribute to ADHD, and their effects are interdependent” [(63), p. 12]. It is both reifying and not in line with empirical evidence to suggest that psychological and social factors merely “influence the disorder itself” [(55), p. 518]. The suggestion that the environment can only amplify or weaken the already present disorder is a misrepresentation of heritability and reifies the construct of ADHD.

## Textual silence

The selective representation of genetic findings also reveals another important reifying mechanism—what is omitted in texts. We refer to this as *textual silence* (64) about important information that would bring a nuanced perspective to ADHD as a construct. We have already discussed the missing heritability problem and the limitations of case-control studies in depth. We will now discuss two other examples that, when unmentioned, can serve to reify ADHD: birth-months studies and the ‘costs of ADHD’ narrative.

### Birth month studies

Scientists worldwide have documented the phenomenon that birth month is a significant risk factor for an ADHD classification [for a review see (65)]. Pupils who are relatively young in their classroom and more likely to display normal age-related impulsivity/inattention, have roughly twice the chance of receiving an ADHD classification and prescribed psychostimulants compared to those who are relatively old in their classroom. This finding demonstrates how social factors can influence who comes to be seen as ‘having’ ADHD. However, there is textual silence toward birth month studies by many scientific authors, which omits an important consideration about the ADHD construct. In a sample of 43 academic textbooks, also used in a study by te Meerman et al. (18), none of the sections on ADHD referred to the phenomenon in relation to ADHD. We view the omission of such a strong correlate that could question the notion of ADHD as a disease entity, to be a passive form of reification.

### The costs of Attention-Deficit/Hyperactivity Disorder

A final reifying mechanism of ADHD concerns the alleged economic burden of ADHD that many studies suggest [see e.g., (66)]. The “costs” of ADHD discourse contains several reifying elements we have discussed earlier. For instance, emphasizing the severity of the problems (e.g., financial burden on society) reifies ADHD. This issue is also an example of circular reasoning as the alleged costs of ADHD often include the costs of the very diagnosis and treatment of the subjective classification. In other words, researchers first argue that certain behaviors are problematic and need diagnosis and treatment, and then submit the costs of this as the very evidence that the behaviors are serious as they also have strong financial implications [e.g. see (67)].

We view the ‘the costs of ADHD-narrative’ primarily as an example of textual silence. The very suggestion that one number can represent the societal costs of unwanted behaviors immediately shifts attention away from a plethora of possible interacting causes of such behaviors and instills silence on motives for classifying children in the first place. These factors include our societal demands and debatable norms, overcrowded classrooms, interests from pharmaceutical companies, poverty, rising divorce rates, bullying, social exclusion, etc. (68, 69). The notion of “costs of ADHD” creates a selective discourse that is mostly silent about complex societal issues or blames children for some of those issues such as divorce and placing “an economic burden on the family” [(70), p. 325].

## Discussion

### Key findings

Our analysis of academic textbooks, scientific articles, websites and videos revealed four ways in which ADHD is reified: language choice, logical fallacies, genetic reductionism and textual silence. Medical jargon and metaphors display ADHD as a disease entity and logical fallacies like generalizing group findings to individuals (ecological fallacy) do the same. Genetic reductionism happens by overstating the results of twin studies while being silent about the disappointing molecular genetic research. Such textual silence also takes place when important information like the relative age effect or the impact of poverty and overcrowded classrooms are not mentioned.

### Limitations

As stated in the method section, this research has mainly focused on identifying discursive mechanisms of reification. This theoretical orientation comes at the expense of the potential of Qualitative Content Analysis (QCA), which is also very suitable for quantification and estimating of, in our case, the prevalence of certain identified mechanisms of reification in a given dataset. This fell outside of our research goal but for potential future studies, we refer to our earlier studies of scientific textbooks [see e.g., (11, 18)]. Furthermore, our theoretical orientation does not consider how the discourse is perceived, for instance in relation to background knowledge and/or the discourse community knowledge consumers reside. Experimental discourse studies would be more suitable for this and are recommended for future research as well.

### The consequences of reification and the need for alternative discourse

Berger and Luckmann (4) state that the result of reification is that “The institutional world, then, is experienced as an objective reality” (p. 77). In the case of ADHD, especially for children this is troublesome because the institutional world “has a history that antedates the individual’s birth” (p. 77). This makes children highly sensitive to accept the questionable notions concerning their alleged condition.

The effects of the reifying discourse surrounding ADHD go far beyond children’s own perception as suffering from a ‘disease.’ How social institutions, such as schools, understand and respond to children rests upon ADHD being constructed, or reified in discourse, as a disorder that some children have and others do not. Through literature such as research articles, textbooks, websites, TV-Programs, and even books for children [see for e.g., (18, 71, 72)] ADHD is reified as an attribute shared across individuals who have harmful brain characteristics. Based on such false assumptions, an institutional world is created that provides medical and school-based interventions and perpetuates the reifying narrative.

The suggestion of dysfunctional brains—based on small group differences, inconsistently found in brain studies of ADHD—and other reifying mechanisms that we have identified in this paper, can contribute to “epistemological violence.” Epistemological violence is what Teo (73) describes as the result when researchers’ interpretations of empirical data construct subjects (the “other”) as inferior or having internal deficits based on objective knowledge, despite alternative interpretations that are equally viable. Nilsson Sjoberg (74) argues that the scientific discourse of ADHD is epistemologically violent in that it “constitutes a totalization of being” (p. 10) in which the unique and diverse characteristics of children are reduced to a brain disorder, and alternative explanations are ignored. Considering how the genetic research is presented as if at odds with environmental influences, and considering the way small correlates concerning brain physiology and anatomy are presented as if applicable to all those with an ADHD classification, the discourse indeed seems totalizing in its effect.

Reification is a necessary mechanism to address when countering discursive practices that result in epistemological violence. The misrepresentation of scientific knowledge, which arises when a heuristic such as ADHD is portrayed as a discrete entity, necessitates that authors who report their own research and the work of others do so with awareness of the pitfalls of reification. It is essential that discourse communities do not repeat harmful errors and habits of writing that can influence how laypeople and professions see children, and how children may come to see themselves. More thoughtful language and logic is needed that can encourage a more transparent approach to discussing the current state, and limitations of scientific knowledge.

### Motives for reification

Berger and Luckmann (4) abstain from making moral, ethical, or intellectual judgments with regard to reification and do not view it as a “cognitive fall from grace” (p. 107), but rather as a modality of human consciousness. Marx on the other hand, founder of the concept of reification, has always been concerned with (unequal) social relations and power, alongside other influences (75). In Marx’ view, reification is not solely the outcome of e.g., rationalization or the use of language but can also be willfully initiated to execute power: reification can be invoked by the powerful who hide behind technical or natural inevitabilities (76). Likewise, the presentation of ADHD as an inherited condition can be instrumental. When reification aids in transferring agency from “ill” behaving individuals to medical specialists who can treat their alleged “sickness,” it is a strong enabler of medicalization. In this context power and control are identified as essential. For instance, studying Hyperkinesis, a predecessor of the ADHD construct, Conrad (77) concluded that “medical social control may be the central issue, as in this role medicine becomes a *de facto* agent of the *status quo*.”

More financial and self-serving motives for medicalization and reification are also subject of longstanding debates, for instance in relation to pharmaceutical “disease mongering” (78). A vivid example is provided by Jutel (79) who describes how lower than average female sexual desire is reified as a disease entity, driven on by drug marketing. Beeker et al. (80) name such efforts as examples of “top-down” forms of psychiatrization.

However, the discourse surrounding medicalization and psychiatrization points to much wider influences. For instance Conrad already in 1975 stated that “the medical profession may not have entirely sought this [medicalizing] role.” And although he adds that “its members have been, in general, disturbingly unconcerned and unquestioning in their acceptance of it” [(77), p. 20], the question remains: what other purposes than power and control do reification and medicalization possibly serve? This leads to a more functionalist sociological approach (81) that is also tentatively considered by Conrad, who asserts that medicalization is functional because it facilitates the individualization of social problems and the depoliticization of deviant behavior.

Such a view makes psychiatry more of a complicit actor in medicalization, but still focuses mostly on “top-down” actors. More contemporary studies on medicalization and psychiatrization discuss discursive practices in which classifications become self-fulfilling prophecies in recursive, discursive “looping” effects (82). These “loops” may be initiated by top-down actors who reify disease-entities, but middle-men and bottom-up actors can help reinforce these classifications and the institutional world that is based on them. Examples are caregivers who help name and personify inner voices in (alleged) cases of multiple personality disorder (82). Additionally, Beeker et al. (83) identify interacting top-down/bottom-up push- and pull-factors in psychiatrization, for example, when people actively solicit certain diagnoses like ADHD to be eligible for (financial) resources and healthcare services, but also when people seek ontological certainty toward their identity and challenges in life (84) or are in search of an “excuse” for their behavior.

The latter begs the underlying question “To the degree that diagnoses and biological explanations do provide an initial excuse for the kind of person one is, the most urgent question is why increasing numbers of people apparently need such an excuse” (85). To answer this question Dehue points to contemporary, neo-liberal “lifestyle politics” that increasingly urge us to be a better version of ourselves, control our impulses and take responsibility for our own actions, while denying our social nature and social embeddedness (86). An in-depth discussion about such cultural tendencies underlying reification and psychiatrization is beyond the scope of this paper. For further analysis of what behavioral norms are reflected by the desired behaviors on the flipside of the unwanted “symptoms” of the ADHD classification, see for example [te Meerman et al. (11), pp. 107–120].

### What to do?

Given a few exceptions, quantitative data on how often these reifying mechanisms occur is lacking. However, given the ease with which flawed information can be found, there seems to be an abundance of misinformation spread around concepts such as ADHD -including through reification in academic textbooks (18, 87) and children books (72). This is alarming, particularly considering ADHD is merely one of over 400 classifications that the DSM 5 currently contains—many likely undergoing similar mechanisms of reification alluding to significant misunderstandings of (ab)normal human behavior. The situation seems urgent enough to warrant practical ways to change current conventions. An example of a possible catalyst of change is a set of guidelines on ADHD psycho-education, recently published in the Netherlands (88). However, this -to our knowledge- first ever published set of guidelines for ADHD-education, is mostly ignored by Dutch mainstream psychiatry. So in addition to changing practices of psycho-education, studying additional mechanisms of reification as well as analyzing the underlying motives of stakeholders and their opposing interests, are necessary to help explain the resistance that the implementation of sound (public) information faces. We hope our study and the set of guidelines it inspired, stimulate both theoretical as well as practical efforts to change the *status quo*.

## Data availability statement

The original contributions presented in this study are included in the references, further inquiries can be directed to the corresponding author.

## Author contributions

SM did the qualitative analysis and wrote the first draft of the manuscript. JF and LB supervised the analysis and commented on the first draft of the manuscript. All authors contributed to manuscript revision, read, and approved the submitted version.
